# Blood pressure–stratified associations of the atherogenic index of plasma with all-cause mortality: a 10-year rural cohort study in China

**DOI:** 10.3389/fcvm.2026.1747068

**Published:** 2026-06-08

**Authors:** Juan Hao, Xiyu Zhao, Chenxi Fan, Mo Lv, Jiahao Wu, Jun Tu, Chunsheng Yang, Xianjia Ning, Yan Li

**Affiliations:** 1 Department of Neurology, Tianjin Medical University General Hospital, Tianjin, China; 2 Department of Critical Care Medicine, Tianjin Jizhou People’s Hospital, Tianjin, China; 3 Institute of Clinical Epidemiology & Evidence-Based Medicine, Tianjin Jizhou People’s Hospital, Tianjin, China; 4 Standardization Office of Palliative Care, Tianjin Jizhou People’s Hospital, Tianjin, China; 5 First Clinical Medical College, Tianjin Medical University, Tianjin, China; 6 Laboratory of Epidemiology, Tianjin Neurological Institute, Tianjin, China; 7 Tianjin Neurological Institute, Key Laboratory of Post-Neuroinjury Neuro-Repair and Regeneration in Central Nervous System, Ministry of Education and Tianjin City, Tianjin, China

**Keywords:** all-cause mortality, atherogenic index of plasma (AIP), hypertension, rural cohort, triglyceride-glucose (TyG) index

## Abstract

**Objective:**

Although the role of AIP in predicting cardiovascular disease has been recognized, its association with all-cause mortality in different blood pressure status populations remains unclear. This study aims to investigate the predictive ability of the Atherogenic Index of Plasma (AIP) and its modified indices for all-cause mortality in rural low-income populations with different blood pressure statuses and compare them with the Triglyceride-Glucose (TyG) index.

**Methods:**

A total of 3,924 rural low-income participants were included in this study. Sociodemographic variables, lifestyle habits, anthropometric measurements, and biochemical markers were systematically recorded. Cox regression and restricted cubic spline (RCS) analyses were used to explore the linear and non-linear associations between TyG and AIP and their modified indices (TyG-BMI, TyG-WHtR, TyG-WC, TyG-WWI, AIP-BMI, AIP-WHtR, AIP-WC, AIP-WWI) and all-cause mortality. Kaplan–Meier survival curves were plotted. Time-dependent receiver operating characteristic (ROC) curves were constructed separately in the hypertensive and non-hypertensive groups to evaluate and compare the predictive ability of TyG and AIP and related indices for all-cause mortality. Additionally, subgroup analyses were performed based on age, gender, and blood glucose status, and mediation analyses were conducted for fasting blood glucose and LDL.

**Results:**

During a median follow-up of 8.82 years, 1,024 cases of all-cause mortality were recorded. After adjusting for confounders, in the hypertensive group, TyG and AIP and their modified indices were significantly negatively associated with all-cause mortality (all *P* < 0.001). Among these, the AIP and its modified index demonstrated stronger protective associations compared to the TyG-based index. Specifically, a one-unit increase in AIP-WHtR (range: −1.47 to 1.68) was associated with a 79% reduction in the risk of all-cause mortality (HR: 0.21, 95% CI: 0.18–0.25, *P* < 0.001); a one-unit increase in AIP (range: −2.41 to 2.90) was associated with a 57% reduction in the risk of all-cause mortality (HR: 0.43, 95% CI: 0.39–0.47, *P* < 0.001). However, in the non-hypertensive group, no significant associations were observed between TyG and AIP and their modified indices and all-cause mortality (all *P* > 0.05). In the hypertensive group, TyG and AIP and their modified indices had a non-linear association with all-cause mortality, characterized by a rapid initial decline followed by a plateau. Compared with TyG and its modified indices, AIP and its modified indices showed better predictive ability. Subgroup analyses further confirmed the robustness of the related conclusions, and no mediating effects of fasting blood glucose and LDL were found.

**Conclusion:**

AIP and its modified indices are significantly negatively associated with all-cause mortality in hypertensive individuals and have a non-linear relationship. No such association was found in the non-hypertensive group. AIP and its modified indices have better predictive ability for all-cause mortality than TyG and its modified indices. These findings provide a scientific basis for the prevention and treatment of cardiovascular diseases in rural areas.

## Introduction

Cardiovascular diseases (CVD) pose a significant global health challenge. According to the Global Burden of Disease (GBD) study, in 2021, the number of people suffering from CVD worldwide reached 612 million, accounting for 26.8% of all deaths, with the heaviest disease burden in regions with low and low-middle Sustainable Development Index (1). It is projected that by 2050, the prevalence of CVD will increase by 90%, mortality will rise by 73.4%, and disability-adjusted life-years (DALYs) will increase by 54.7% (2).

In recent years, the association of insulin resistance (IR) and the Atherogenic Index of Plasma (AIP) with CVD has attracted widespread attention. The Triglyceride-Glucose (TyG) index is an effective indicator for assessing insulin resistance. Pathophysiological studies have shown that insulin resistance promotes inflammation, endothelial dysfunction, and dyslipidemia, which may be the main mechanisms underlying the progression of coronary heart disease (3). The Atherogenic Index of Plasma (AIP) is a novel parameter for evaluating plasma atherogenicity and is profoundly linked to atherosclerotic burden and the occurrence of CVD (4, 5).

Previous studies have shown that the cumulative TyG index is associated with an increased risk of CVD, with a more significant increase in risk related to longer exposure to a higher TyG index (6). Both the TyG index and AIP are significantly associated with the occurrence of coronary heart disease, with AIP showing a more pronounced association with cardiovascular disease severity in patients with normal glucose regulation (7). The TyG index appears to be associated with an increased risk of adverse cardiovascular events, all-cause mortality, non-fatal myocardial infarction, and revascularization in patients with coronary heart disease (8). Moreover, obesity is also a direct risk factor for the occurrence, development, and mortality of CVD and can lead to a variety of problems, including dyslipidemia, type 2 diabetes, hypertension, and sleep disorders (9). Research results have shown that abdominal obesity is significantly associated with cardiovascular events in people with diabetes (10). The visceral adiposity index has high predictive value for all-cause, cardiovascular, and cancer mortality risks (11).

Hypertension is the most important risk factor for CVD (stroke and heart disease), with a nearly logarithmic linear relationship between blood pressure levels and cardiovascular mortality risk in middle- and older-aged individuals (12). Hypertension contributes to the development of CVD through pathophysiological processes such as endothelial dysfunction, vascular inflammation, increased arterial stiffness, and reduced distensibility (13). However, although studies have separately explored the relationships of obesity indices, the TyG index, and AIP with CVD, research combining these indices for analysis is still relatively limited. Moreover, the associations of TyG and AIP with mortality in populations with different blood pressure statuses remain uncertain, especially in rural areas. Rural areas face a more severe situation in the prevention and control of CVD due to relatively scarce medical resources and weaker health awareness. Therefore, this study aims to investigate the associations of combined analysis of the AIP index, TyG, and obesity indices with mortality in populations with different blood pressure statuses through a 10-year rural cohort study and to compare the predictive roles of TyG- and AIP-related indices, in order to provide a scientific basis for the prevention and treatment of CVD in rural areas.

## Methods

### Study design

This population-based cross-sectional survey was conducted in rural areas of Tianjin, China. Participants came from the Tianjin Brain Research Project. The study population comprised 14,251 participants from 18 administrative villages in rural Tianjin, approximately 95% of whom were low-income farmers with an annual per capita income below $100 in 1991 and below $1,000 in 2010. In addition to these income-based indicators, we utilized the multidimensional poverty index (MPI) to provide a more comprehensive definition of low-income status. MPI was calculated based on the Alkire-Foster method, which considers three key dimensions: education, health, and living standards. This multidimensional approach considers factors beyond income, including education, health, and living standards, and further validates the classification of rural residents as low-income compared to their urban counterparts. According to 2014 national statistical data, the MPI for rural residents was 0.028 compared to 0.007 for urban residents, underscoring the greater deprivation experienced by rural populations. This study incorporated cohort data from 2014 to 2023, with a total of 9,219 participants at baseline. After screening, 4,113 individuals with missing baseline TyG and AIP data (due to missing TG, HDL-C, or fasting blood glucose measurements) and 1,182 individuals lacking the baseline variable data required for this study (including age and gender) were excluded, resulting in a final sample of 3,924 participants: 2,662 in the hypertension group and 1,262 in the non-hypertension group. Since this study aimed to investigate all-cause mortality among low-income rural populations, participants with cardiovascular disease at baseline were not excluded. Thanks to the three-tier mortality reporting system, no loss to follow-up occurred during the median follow-up period of 8.82 years. The present analysis is a pre-specified secondary analysis of the Tianjin Brain Research. The parent study and all subsequent observational analyses were approved by the Ethics Committee of Tianjin Medical University General Hospital (Approval No. IRB2018-100-02) and was conducted in accordance with the Declaration of Helsinki. All participants provided written informed consent at enrollment, which explicitly covered the use of their data for subsequent observational analyses.

### Data collection

Between 2014 and 2023, trained interviewers collected sociodemographic and clinical data through face-to-face questionnaires, recording gender, age, and history of diabetes and hypertension diagnoses. Lifestyle variables included current smoking and alcohol consumption status. Trained medical staff measured height, weight, waist circumference, and blood pressure using calibrated instruments. Blood pressure was measured with a mercury sphygmomanometer, and the same observer recorded the average of two to three readings of systolic blood pressure (SBP) and diastolic blood pressure (DBP) to reduce systematic bias. After fasting for at least 12 h, blood samples were collected in the morning to test fasting blood glucose, triglycerides, total cholesterol, high-density lipoprotein cholesterol (HDL-C), and low-density lipoprotein cholesterol (LDL-C). Mortality data were collected through a three-tier reporting cascade: village physicians reported monthly deaths via the township public-health network for entry into the district mortality database; the township preventive-care office quarterly consolidated records, amended omissions or errors, and cross-checked them against household-registry cancellations; the research team then conducted annual village-by-household reverification, with any discrepant or missing cases resolved by two independent reviewers who retrieved the original death certificate, village logbook, or hospital chart and, when necessary, performed a second home visit to fix the exact date and cause of death.

### Variable definitions

Body mass index (BMI) was calculated as weight (kilograms) divided by height (meters) squared (14). Smoking status was defined as individuals who smoked at least one cigarette per day for more than 1 year; alcohol consumption was considered positive if the intake of ethanol was 45 g or more per day in the previous year (15). Hypertension was defined as having a systolic blood pressure of ≥140 mmHg or a diastolic blood pressure of ≥90 mmHg, current use of antihypertensive medication, or self-reported physician-diagnosed hypertension (16). Diabetes was defined according to the American Diabetes Association criteria: glycated hemoglobin A1C ≥ 6.5%, fasting plasma glucose ≥126 mg/dL, 2-hour oral glucose tolerance test value ≥200 mg/dL, use of antidiabetic medication, or self-reported diabetes (17).

The TyG index was calculated using the formula TyG = ln[TG (mg/dL) × FBG (mg/dL)/2] (18). AIP was calculated using the formula AIP = log10[TG (mmol/L)/HDL-C (mmol/L)] (19). Waist-to-height ratio (WHtR) was defined as waist circumference divided by height (15). Waist-to-weight index (WWI) was calculated as waist circumference (cm) divided by weight (kg) squared (20). Composite indices of TyG and AIP were generated by multiplying the TyG and AIP indices with BMI, WHtR, waist circumference, and WWI, respectively. The composite indicators developed in this study are all continuous variables, generated by multiplying the corresponding component indicators, and thus belong to dimensionless metrics. The interpretation of the effect magnitude for “a one-unit increment” must strictly rely on the observed distribution characteristics. Taking the hypertension group as an example, the observed distribution range of AIP-WHtR spans from−1.47 to 1.68; a one-unit increment accounts for approximately one-third of this full range rather than representing minor fluctuations.

### Statistical methods

Continuous variables are presented as mean ± standard deviation, and categorical variables as counts (%). Univariate Cox regression analysis was used to identify factors associated with all-cause mortality, and multivariate Cox regression analysis was performed to examine the multivariate associations of TyG and AIP and related indices with all-cause mortality, with results presented as hazard ratios (HR) and 95% confidence intervals (CI). Kaplan–Meier survival curves were plotted for quartiles of the independent variables. Restricted cubic spline (RCS) regression analysis was implemented using the rms (Regression Modeling Strategies) package in R to assess potential non-linear relationships between independent variables and outcomes. The area under the receiver operating characteristic (ROC) curves for each index's predictive ability for the outcome was calculated separately for the hypertensive and non-hypertensive groups to compare the predictive power of each index. The mediation effect analysis was conducted using the mediation analysis package in R, with AIP as the exposure variable, all-cause mortality as the outcome variable, and FBG or LDL-C as candidate mediation variables. The mean causal mediation effect, mean direct effect, and mediation proportion were estimated using the quasi-Bayesian Monte Carlo method (1,000 simulations), with 95% confidence intervals derived from the corresponding percentile distribution. The model was adjusted for factors such as gender, age, years of education, smoking history, and alcohol consumption history. All analyses were completed using SPSS 25.0 and R software. All statistical tests were two-sided, and a *P* value <0.05 was considered statistically significant.

## Results

### Demographic characteristics

**Figure 1:**
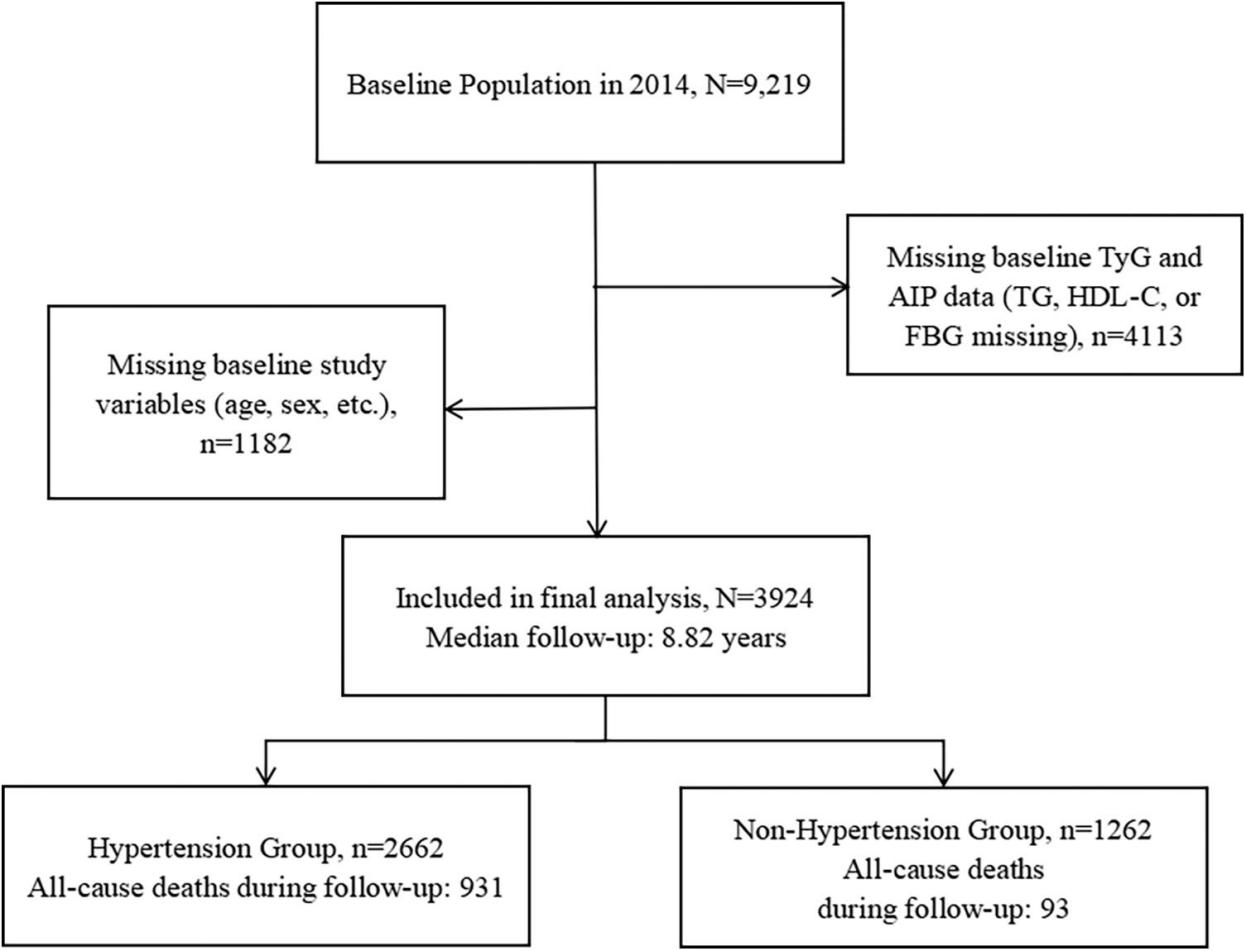
Flowchart of participant selection. Figure illustrates the process of participant selection from initial screening to final inclusion in the study, including exclusion criteria and the final sample size. TyG, triglyceride-glucose index; AIP, atherogenic index of plasma; TG, triglycerides; HDL-C, high-density lipoprotein cholesterol; FBG, fasting blood glucose.

A total of 3,924 participants were included in this study, with a mean follow-up duration of 8.82 years (Figure 1). There were 1,632 men and 2,292 women, with a mean age of 60.14 years. The mean TyG index for the overall population was 8.83, 8.73 for men and 8.90 for women; the mean AIP index was 0.06, 0.01 for men and 0.10 for women. There were 2,662 participants with hypertension, including 1,078 men and 1,584 women. During the follow-up period, 1,024 participants died, with 485 men and 539 women (Table 1).

**Table 1:** Characteristics of participants.

Characteristics	Men (*n* = 1,632)	Women (*n* = 2,292)	Total (*n* = 3,924)
Follow-up duration, years, means ± SD	8.68 ± 2.45	8.93 ± 2.32	8.82 ± 2.38
Age, years, means ± SD	61.35 ± 9.95	59.27 ± 9.45	60.14 ± 9.71
Years of education, years, means ± SD	6.39 ± 2.95	4.31 ± 3.68	5.17 ± 3.54
FBG, mmol/L, means ± SD	5.93 ± 1.42	5.94 ± 1.67	5.94 ± 1.60
TG, mmol/L, means ± SD	1.60 ± 3.33	1.87 ± 1.17	1.76 ± 1.25
TC, mmol/L, means ± SD	4.60 ± 0.99	5.04 ± 1.09	4.86 ± 1.08
LDL-C, mmol/L, means ± SD	2.53 ± 0.83	2.74 ± 0.92	2.65 ± 0.89
HDL-C, mmol/L, means ± SD	1.39 ± 0.45	1.50 ± 0.47	1.45 ± 0.47
Smoking, *n* (%)			
Never smoking	278 (11.8)	2,070 (88.2)	2,348 (100)
Current smoking	812 (93.2)	59 (6.8)	871 (100)
Ever smoking	523 (85.7)	87 (14.3)	610 (100)
Alcohol consumption, *n* (%)			
Never drinking	464 (17.6)	2,177 (82.4)	2,641 (100)
Current drinking	790 (94.4)	47 (5.6)	837 (100)
Ever drinking	365 (88.0)	50 (12.0)	415 (100)
Hypertension, *n* (%)	1,078 (40.5)	1,584 (59.5)	2,662 (100)
Diabetes, *n* (%)	298 (38.5)	477 (61.5)	775 (100)
Death, *n* (%)	485 (47.4)	539 (52.6)	1,024 (100)
BMI, kg/m^2^, means ± SD	25.16 ± 3.40	25.86 ± 3.79	25.57 ± 3.65
WC, cm, means ± SD	89.47 ± 8.93	89.11 ± 9.08	89.26 ± 9.02
WHtR, means ± SD	0.54 ± 0.05	0.57 ± 0.06	0.56 ± 0.06
WWI, means ± SD	10.74 ± 0.62	11.32 ± 0.73	11.08 ± 0.75
TyG, means ± SD	8.73 ± 0.63	8.90 ± 0.62	8.83 ± 0.63
TyG-BMI, means ± SD	220.42 ± 38.67	230.90 ± 41.27	226.54 ± 54.68
TyG-WHtR, means ± SD	4.71 ± 0.65	5.13 ± 0.72	4.95 ± 0.72
TyG-WC, means ± SD	783.11 ± 112.16	795.12 ± 112.18	790.13 ± 112.31
TyG-WWI, means ± SD	93.80 ± 9.20	100.88 ± 10.35	97.94 ± 10.49
AIP, means ± SD	0.01 ± 0.73	0.10 ± 0.70	0.06 ± 0.71
AIP-BMI, means ± SD	1.29 ± 18.69	3.40 ± 18.28	2.52 ± 18.48
AIP-WHtR, means ± SD	0.02 ± 0.39	0.07 ± 0.41	0.05 ± 0.40
AIP-WC, means ± SD	3.57 ± 65.78	10.92 ± 62.86	7.87 ± 64.19
AIP-WWI, means ± SD	0.18 ± 7.80	1.19 ± 7.96	0.77 ± 7.91

BMI, body mass index; WC, waist circumference; WHtR, waist-to-height ratio; WWI, weight-adjusted waist index; TyG, triglyceride-glucose index; AIP, atherogenic index of plasma; FBG, fasting blood glucose; TG, triglycerides; TC, total cholesterol; LDL-C, low-density lipoprotein cholesterol; HDL-C, high-density lipoprotein cholesterol.

### Survival analysis

Univariate Cox regression analysis showed that in the hypertensive group, sex, age, years of education, triglycerides (TG), total cholesterol (TC), low-density lipoprotein cholesterol (LDL-C), high-density lipoprotein cholesterol (HDL-C), smoking history, alcohol consumption history, BMI, waist circumference (WC), waist-to-height ratio (WHtR), waist-to-weight index (WWI), TyG and AIP and related indices were associated with all-cause mortality (all *P* < 0.05). In the non-hypertensive group, sex, age, years of education, fasting blood glucose (FBG), smoking history, alcohol consumption history, history of diabetes, BMI, WWI, TyG-BMI, TyG-WWI were associated with all-cause mortality (all *P* < 0.05) (Table 2). After adjusting for relevant confounding factors, in model 2 (adjusted for sex, age, years of education) and model 3 (adjusted for sex, age, years of education, smoking history, alcohol consumption history), TyG and AIP and related indices were significantly associated with all-cause mortality in the hypertensive group (all *P* < 0.001). Specifically, a one-unit increase in AIP-WHtR was associated with a 79% reduction in the risk of all-cause mortality (HR: 0.21, 95% CI: 0.18–0.25, *P* < 0.001); a one-unit increase in AIP was associated with a 57% reduction in the risk of all-cause mortality (HR: 0.43, 95% CI: 0.39–0.47, *P* < 0.001). However, in the non-hypertensive group, no significant associations were observed between TyG and AIP and related indices and all-cause mortality (all *P* > 0.05) (Table 3).

**Table 2:** Univariate Cox regression results for all-cause mortality by blood pressure status.

Characteristics	Reference	Hypertensive group	*P*	Non-hypertensive group	*P*
		HR (95% CI)		HR (95% CI)	
Female	Male	0.76 (0.67, 0.87)	<0.001	0.51 (0.34, 0.77)	0.001
Age		1.06 (1.05, 1.06)	<0.001	1.13 (1.11, 1.16)	<0.001
Years of education		0.94 (0.93, 0.96)	<0.001	0.87 (0.82, 0.92)	<0.001
FBG		1.00 (0.96, 1.04)	0.924	1.29 (1.20, 1.39)	<0.001
TG		0.47 (0.42, 0.51)	<0.001	0.81 (0.65, 1.03)	0.081
TC		0.90 (0.84, 0.95)	<0.001	0.98 (0.81, 1.20)	0.846
LDL-C		0.92 (0.85, 0.99)	0.028	1.04 (0.82, 1.31)	0.774
HDL-C		1.65 (1.53, 1.79)	<0.001	0.90 (0.56, 1.43)	0.652
Smoking	Never smoking				
Current smoking		1.24 (1.05, 1.45)	0.010	1.45 (0.91, 2.31)	0.122
Ever smoking		1.41 (1.19, 1.66)	<0.001	2.01 (1.17, 3.44)	0.011
Alcohol consumption	Never drinking				
Current drinking		1.03 (0.88, 1.22)	0.683	0.58 (0.31, 1.11)	0.099
Ever drinking		1.44 (1.19, 1.74)	<0.001	2.92 (1.81, 4.71)	<0.001
Diabetes		0.93 (0.80, 1.08)	0.337	4.19 (2.70, 6.49)	<0.001
BMI		0.92 (0.90, 0.93)	<0.001	0.87 (0.81, 0.92)	<0.001
WC		0.98 (0.97, 0.99)	<0.001	0.98 (0.96, 1.01)	0.132
WHtR		0.08 (0.03, 0.24)	<0.001	0.38 (0.01, 14.94)	0.606
WWI		1.15 (1.06, 1.26)	0.002	1.56 (1.24, 1.96)	<0.001
TyG		0.43 (0.39, 0.48)	<0.001	1.02 (0.73, 1.42)	0.922
TyG-BMI		0.99 (0.99, 0.99)	<0.001	0.99 (0.98, 1.00)	<0.001
TyG-WHtR		0.59 (0.54, 0.65)	<0.001	0.94 (0.69, 1.28)	0.693
TyG-WC		1.00 (1.00, 1.00)	<0.001	1.00 (1.00, 1.00)	0.279
TyG-WWI		0.97 (0.97, 0.98)	<0.001	1.02 (1.00, 1.04)	0.018
AIP		0.41 (0.38, 0.45)	<0.001	0.83 (0.61, 1.11)	0.206
AIP-BMI		0.97 (0.96, 0.97)	<0.001	0.99 (0.98, 1.01)	0.258
AIP-WHtR		0.20 (0.17, 0.23)	<0.001	0.66 (0.38, 1.16)	0.147
AIP-WC		0.99 (0.99, 0.99)	<0.001	1.00 (0.99, 1.00)	0.171
AIP-WWI		0.92 (0.92, 0.93)	<0.001	0.98 (0.95, 1.01)	0.115

HR, hazard ratio; CI, confidence interval; TyG, triglyceride-glucose index; AIP, atherogenic index of plasma; BMI, body mass index; WC, waist circumference; WHtR, waist-to-height ratio; WWI, weight-adjusted waist index; FBG, fasting blood glucose; TG, triglycerides; TC, total cholesterol; LDL-C, low-density lipoprotein cholesterol; HDL-C, high-density lipoprotein cholesterol.

**Table 3:** Multivariate Cox regression results for TyG, AIP, and related derived indices with all-cause mortality by blood pressure status.

Characteristics	Model 1	*P*	Model 2	*P*	Model 3	*P*
	HR (95% CI)		HR (95% CI)		HR (95% CI)	
Hypertensive group:						
TyG	0.43 (0.39, 0.48)	<0.001	0.45 (0.40, 0.50)	<0.001	0.44 (0.40, 0.50)	<0.001
TyG-BMI	0.99 (0.99, 0.99)	<0.001	0.99 (0.99, 0.99)	<0.001	0.99 (0.99, 0.99)	<0.001
TyG-WHtR	0.59 (0.54, 0.65)	<0.001	0.59 (0.54, 0.66)	<0.001	0.59 (0.53, 0.65)	<0.001
TyG-WC	1.00 (1.00, 1.00)	<0.001	1.00 (1.00, 1.00)	<0.001	1.00 (1.00, 1.00)	<0.001
TyG-WWI	0.97 (0.97, 0.98)	<0.001	0.96 (0.96, 0.97)	<0.001	0.96 (0.96, 0.97)	<0.001
AIP	0.41 (0.38, 0.45)	<0.001	0.44 (0.40, 0.48)	<0.001	0.43 (0.39, 0.47)	<0.001
AIP-BMI	0.97 (0.96, 0.97)	<0.001	0.97 (0.96, 0.97)	<0.001	0.97 (0.96, 0.97)	<0.001
AIP-WHtR	0.20 (0.17, 0.23)	<0.001	0.22 (0.19, 0.26)	<0.001	0.21 (0.18, 0.25)	<0.001
AIP-WC	0.99 (0.99, 0.99)	<0.001	0.99 (0.99, 0.99)	<0.001	0.99 (0.99, 0.99)	<0.001
AIP-WWI	0.92 (0.92, 0.93)	<0.001	0.93 (0.92, 0.94)	<0.001	0.93 (0.92, 0.93)	<0.001
Non-hypertensive group:						
TyG	1.02 (0.73, 1.42)	0.922	1.21 (0.83, 1.78)	0.327	1.11 (0.75, 1.65)	0.597
TyG-BMI	0.99 (0.98, 1.00)	<0.001	1.00 (0.99, 1.00)	0.525	1.00 (0.11, 1.01)	0.549
TyG-WHtR	0.94 (0.69, 1.28)	0.693	0.95 (0.67, 1.33)	0.744	0.95 (0.68, 1.33)	0.761
TyG-WC	1.00 (1.00, 1.00)	0.279	1.00 (0.11, 1.00)	0.455	1.00 (1.00, 1.00)	0.498
TyG-WWI	1.02 (1.00, 1.04)	0.018	1.01 (0.98, 1.03)	0.683	1.00 (0.98, 1.03)	0.774
AIP	0.83 (0.61, 1.11)	0.206	0.96 (0.70, 1.30)	0.771	0.87 (0.64, 1.19)	0.389
AIP-BMI	0.99 (0.98, 1.01)	0.258	1.00 (0.99, 1.01)	0.864	1.00 (0.98, 1.01)	0.489
AIP-WHtR	0.66 (0.38, 1.16)	0.147	0.89 (0.50, 1.59)	0.696	0.76 (0.42, 1.36)	0.353
AIP-WC	1.00 (0.99, 1.00)	0.171	1.00 (1.00, 1.00)	0.763	1.00 (1.00, 1.00)	0.392
AIP-WWI	0.98 (0.95, 1.01)	0.115	0.99 (0.97, 1.02)	0.630	0.99 (0.96, 1.01)	0.310

Model 1: unadjusted. Model 2: adjusted for sex, age, and years of education. Model 3: adjusted for sex, age, years of education, smoking history, and alcohol consumption history. HR, hazard ratio; CI, confidence interval; TyG, triglyceride-glucose index; AIP, atherogenic index of plasma; BMI, body mass index; WC, waist circumference; WHtR, waist-to-height ratio; WWI, weight-adjusted waist index.

**Figure 2:**
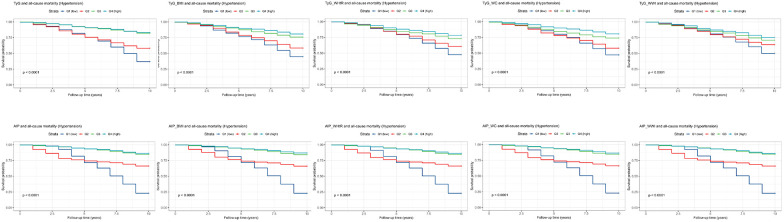
Kaplan–Meier survival curves for all-cause mortality in the hypertensive group. Figure shows the Kaplan–Meier survival curves for all-cause mortality by quartiles of TyG and AIP-related indices in the hypertensive group, reflecting differences in survival rates over time. TyG, triglyceride-glucose index; AIP, atherogenic index of plasma; BMI, body mass index; WC, waist circumference; WHtR, waist-to-height ratio; WWI, weight-adjusted waist index.

**Figure 3:**
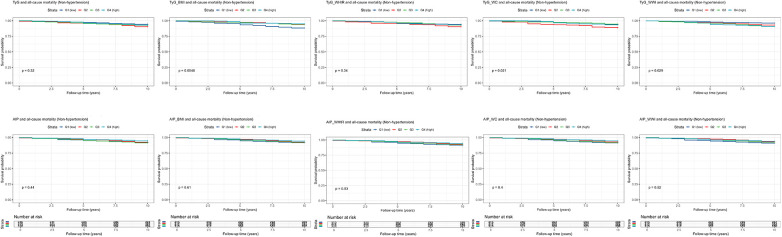
Kaplan–Meier survival curves for all-cause mortality in the non-hypertensive group. Figure shows the Kaplan–Meier survival curves for all-cause mortality by quartiles of TyG and AIP-related indices in the non-hypertensive group, reflecting differences in survival rates over time. TyG, triglyceride-glucose index; AIP, atherogenic index of plasma; BMI, body mass index; WC, waist circumference; WHtR, waist-to-height ratio; WWI, weight-adjusted waist index.

Figures 2, 3 show the Kaplan–Meier survival curves for all-cause mortality by quartiles of TyG and AIP-related indices in participants in the hypertensive and non-hypertensive groups, respectively. The number of participants at each follow-up time point shown in the survival curve is presented in Supplementary Tables S1 and S2. For each TyG and AIP-related indicator, participants were divided into four quartiles based on the distribution of the indicator across different groups [Q1 = minimum value to P25, Q2 = P25–P50 (median), Q3 = P50–P75, and Q4 = P75 to maximum value]: Q1 represents the lowest quartile, and Q4 represents the highest quartile. The specific cutoff values for each quartile are also listed in Supplementary Table S3.

### RCS analysis results

**Figure 4:**
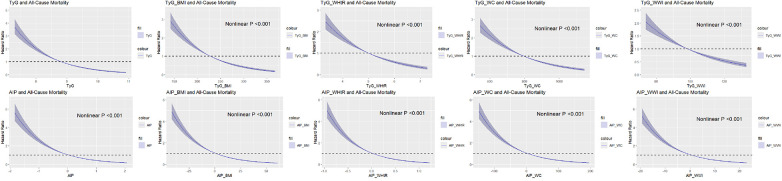
RCS analysis of all-cause mortality for TyG and AIP-related indices in the hypertensive group. Figure presents the results of restricted cubic spline (RCS) analysis of the non-linear relationships between TyG and AIP-related indices and all-cause mortality in the hypertensive group. RCS, restricted cubic spline; TyG, triglyceride-glucose index; AIP, atherogenic index of plasma; BMI, body mass index; WC, waist circumference; WHtR, waist-to-height ratio; WWI, weight-adjusted waist index.

**Figure 5:**
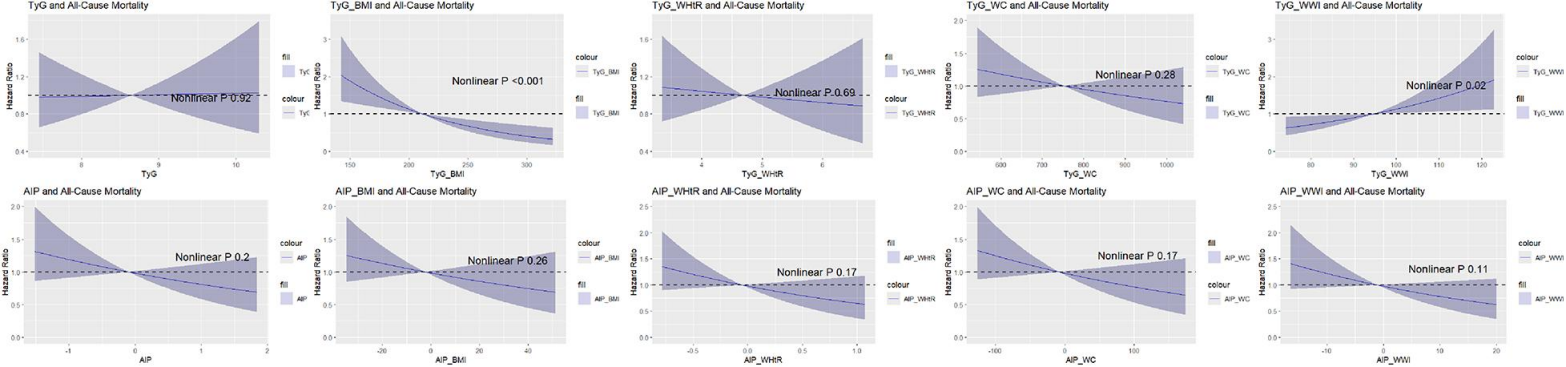
RCS analysis of all-cause mortality for TyG and AIP-related indices in the non-hypertensive group. Figure presents the results of restricted cubic spline (RCS) analysis of the non-linear relationships between TyG and AIP-related indices and all-cause mortality in the non-hypertensive group. RCS, restricted cubic spline; TyG, triglyceride-glucose index; AIP, atherogenic index of plasma; BMI, body mass index; WC, waist circumference; WHtR, waist-to-height ratio; WWI, weight-adjusted waist index.

We performed RCS analysis of the associations between TyG and AIP and related indices and all-cause mortality in the hypertensive and non-hypertensive groups (Figures 4, 5). The results showed that in the hypertensive group, there were significant non-linear associations between TyG and AIP and related indices and all-cause mortality (all *P* < 0.001), with the risk of all-cause mortality rapidly decreasing and then stabilizing as the independent variables increased. In the non-hypertensive group, only TyG-BMI and TyG-WWI had non-linear associations with all-cause mortality (both *P* < 0.05). Specifically, the risk of all-cause mortality rapidly decreased and then stabilized with increasing TyG-BMI; the all-cause mortality rate slowly increased and then rapidly increased with increasing TyG-WWI.

### ROC analysis results

**Figure 6:**
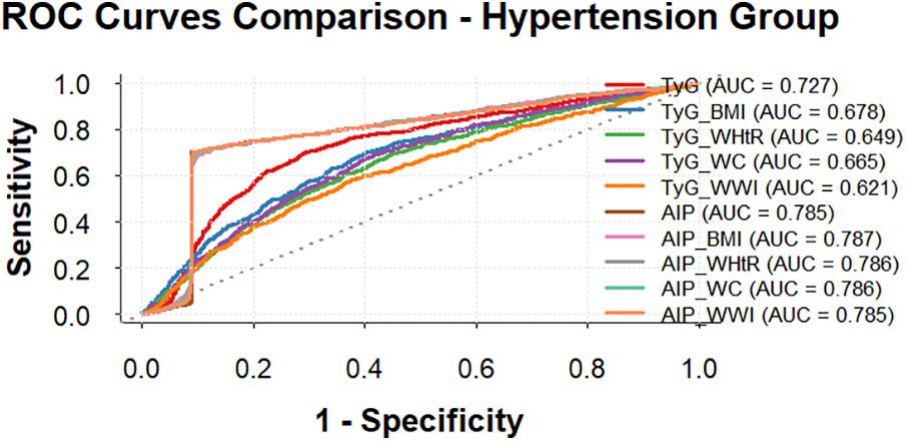
Time-dependent ROC curves for predictive ability of TyG and AIP-related indices in the hypertensive group. Figure shows the time-dependent receiver operating characteristic (ROC) curves for the predictive ability of TyG and AIP-related indices for all-cause mortality in the hypertensive group, reflecting the predictive power of each index. ROC, receiver operating characteristic; AUC, area under the curve; TyG, triglyceride-glucose index; AIP, atherogenic index of plasma; BMI, body mass index; WC, waist circumference; WHtR, waist-to-height ratio; WWI, weight-adjusted waist index.

**Figure 7:**
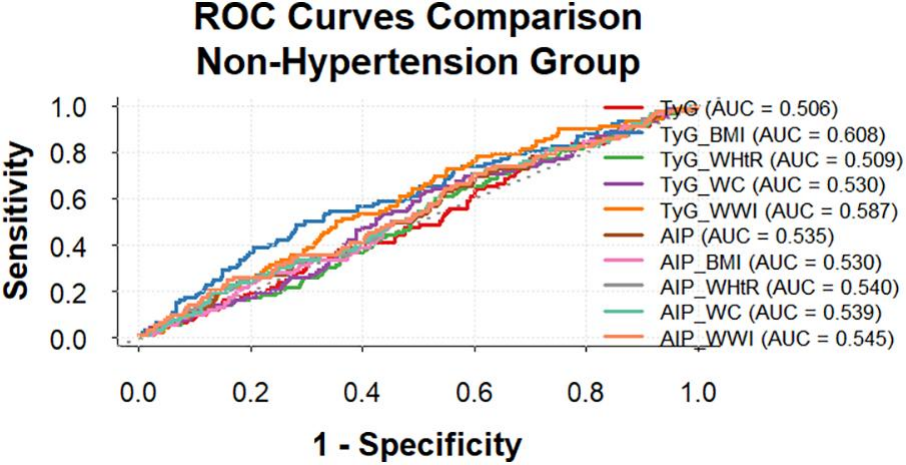
Time-dependent ROC curves for predictive ability of TyG and AIP-related indices in the non-hypertensive group. Figure shows the time-dependent receiver operating characteristic (ROC) curves for the predictive ability of TyG and AIP-related indices for all-cause mortality in the non-hypertensive group, reflecting the predictive power of each index. ROC, receiver operating characteristic; AUC, area under the curve; TyG, triglyceride-glucose index; AIP, atherogenic index of plasma; BMI, body mass index; WC, waist circumference; WHtR, waist-to-height ratio; WWI, weight-adjusted waist index.

We conducted time-dependent ROC curve analysis of the associations between TyG and AIP and related indices and all-cause mortality separately in the hypertensive and non-hypertensive groups, comparing the predictive ability of each independent variable in each group by the area under the curve (see Figures 6, 7). The results showed that compared with the non-hypertensive group, TyG and AIP and related indices had stronger predictive ability for all-cause mortality in the hypertensive group. In the hypertensive group, AIP and related indices had stronger predictive ability than TyG and related indices, with AIP-BMI having the strongest predictive ability (AUC = 0.787), and AIP-WHtR and AIP-WC also showing strong predictive ability (AUC = 0.786).

### Mediation analysis

**Figure 8:**
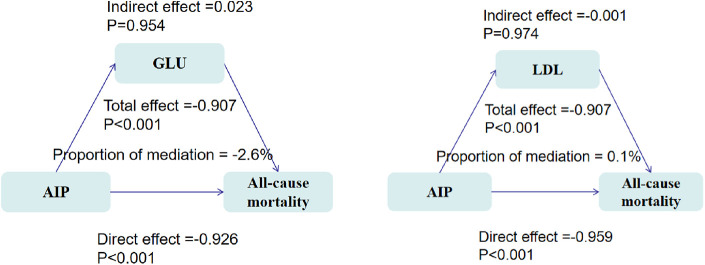
Mediation analysis of the association between AIP and all-cause mortality in the hypertensive group. Figure presents the results of mediation analysis of the association between AIP and all-cause mortality in the hypertensive group, showing that fasting blood glucose and LDL did not mediate the association. AIP, atherogenic index of plasma; FBG, fasting blood glucose; LDL-C, low-density lipoprotein cholesterol; CI, confidence interval.

We conducted mediation analysis in the hypertension group. Neither fasting blood glucose (FBG) nor low-density lipoprotein cholesterol (LDL-C) significantly mediated the association between AIP and all-cause mortality (both *P*-values >0.05) (Figure 8). This absence of mediation suggests that in our study cohort, the relationship between AIP and mortality in hypertensive patients is not explained by pathways driven by blood glucose or LDL-C.

### Subgroup analysis

We performed subgroup analyses based on sex, age, and blood glucose status (Supplementary Tables S4–S6). The results showed that in each subgroup, TyG and AIP and related indices were significantly associated with all-cause mortality in the hypertensive population (all *P* < 0.001). Except for TyG being significantly positively associated with all-cause mortality in non-hypertensive individuals aged <60 years (*P* = 0.016), no significant associations were found between TyG and AIP and related indices and all-cause mortality in other subgroups of non-hypertensive individuals (all *P* > 0.05).

Given the gender imbalance in this cohort (58% female), we conducted sex-stratified analyses and interaction tests. For AIP, both sexes in the hypertension group showed significant associations with all-cause mortality (both *P* < 0.001), but the interaction test revealed statistically significant gender differences (*P* for interaction <0.001): the protective effect was stronger in females (HR 0.38 vs. male HR 0.49). In the non-hypertension group, the AIP interaction was not significant (*P* = 0.388).

## Discussion

This study is the first to investigate the relationship between AIP and TyG and related indices and all-cause mortality in individuals with different blood pressure statuses. The results show that AIP and TyG and related indices are significantly negatively associated with all-cause mortality in hypertensive individuals and have a non-linear relationship. However, no such association was found in the non-hypertensive group. Additionally, AIP and related indices have a higher predictive ability for all-cause mortality than TyG and related indices. In the hypertensive group, the association between AIP and mortality was stronger in females than in males (interaction *P* < 0.001). This aligns with the gender dimorphism in lipid metabolism—females typically exhibit higher HDL-C levels, which may amplify the predictive signal of AIP (based on TG/HDL-C). Rather than acting as a mediator, blood pressure status appears to function as an effect modifier of the AIP–mortality association: the prognostic value of AIP and its modified indices is concentrated in the hypertensive stratum and is essentially absent in the non-hypertensive stratum. The non-significant mediation through FBG and LDL-C should therefore not be interpreted as a mechanistic pathway, but rather as an indication that the AIP signal in hypertension is independent of these two routine biochemical mediators.

The TyG index, as an effective indicator for assessing insulin resistance, has been confirmed by numerous studies to be associated with mortality and is modulated by blood pressure levels. A prospective study of 6,245 individuals showed that the combination of low TyG index and low systolic blood pressure levels is associated with a reduced risk of all-cause and cardiovascular mortality, but this association disappears with increasing blood pressure (21). Another study on hypertensive patients showed a J-shaped dose-response relationship between the TyG index and cardiovascular and all-cause mortality in hypertensive patients (22). Moreover, a study on patients with essential hypertension showed that a higher TyG index is associated with increased 30, 60, and 90 days all-cause mortality risks in ICU patients with essential hypertension (23). Zhou et al. showed that the association between the triglyceride-glucose index (TyG) and cardiovascular/all-cause mortality in hypertensive patients is non-linear, with both high and low TyG levels increasing mortality risk in hypertensive patients (24). Insulin resistance induces oxidative stress, activates the sympathetic nervous system, impairs endothelial function, and leads to hyperglycemia or dyslipidemia, thereby causing systemic target organ damage (25–28). Insulin resistance also exacerbates the condition of hypertensive patients through membrane ion exchange, inhibition of natriuretic peptide activity, sodium retention, and plasma volume expansion, significantly increasing their mortality risk (23, 29). Moreover, the state of insulin resistance is associated with abnormalities in various cardiovascular metabolic factors, including elevated plasminogen activator inhibitor-1 (PAI-1), elevated fibrinogen, and increased platelet viscosity, all of which are important cardiovascular risk factors (30, 31).Our study found that TyG and related indices are significantly negatively associated with all-cause mortality risk in hypertensive individuals and have a non-linear relationship. However, no such association was found in the non-hypertensive group. This may be related to the pathophysiological mechanisms of insulin resistance in hypertensive patients. This BP-specific pattern is biologically plausible: in hypertension, insulin resistance acts in synergy with chronically elevated arterial pressure to amplify endothelial dysfunction, sympathetic over-activation, sodium retention and vascular remodeling, so that even modest changes in glucose-lipid metabolism translate into measurable mortality differences. In normotensive individuals, in whom these pressure-driven amplifying pathways are not engaged, the same metabolic perturbations exert a much smaller absolute effect on mortality, which likely explains the absence of an association in the non-hypertensive stratum.

AIP is an inexpensive and easily assessed marker that can be used to evaluate the progression of atherosclerosis (32). AIP has been widely shown to be associated with the risk of developing cardiovascular disease, but studies on its association with all-cause mortality are limited, and its predictive role for death in populations with different blood pressure statuses has not been reported. A study of 14,063 US adults found a U-shaped non-linear association between AIP and all-cause mortality (33). Elevated AIP levels are closely related to higher mortality risks in patients with cardiovascular-kidney-metabolic (CKM) syndrome, especially late-stage all-cause mortality and non-late-stage and late-stage CVD mortality (34). A study on adult hypertensive patients showed that AIP is associated with all-cause mortality and cardiovascular disease-specific mortality in hypertensive patients, with both low and high AIP indices increasing mortality in hypertensive patients (35). Qian et al. analyzed the China Health and Retirement Longitudinal Study (CHARLS) cohort and found that AIP and TyG both have predictive effects on post-stroke mortality, with higher AIP associated with lower mortality risk and a chain-like mediating effect with TyG (36). The potential mechanism underlying the association between AIP and cardiovascular mortality can be explained by the association of this index with lipoprotein particle size: it is negatively correlated with LDL cholesterol particle size (37). AIP values are closely related to fractional esterification rate of HDL-C [FER(HDL-C)] values and lipoprotein particle size and can therefore be used as a marker of plasma atherosclerosis (19).

Compared with traditional lipid ratios such as non-HDL-C and TC/HDL-C, AIP integrates triglyceride and HDL-C information through a logarithmic transformation that more directly reflects atherogenic lipoprotein particle size and esterification activity, and may therefore better capture the residual atherogenic risk that persists after conventional lipid targets are met. Our finding that AIP-based indices outperform TyG-based indices in hypertensive individuals supports the concept that, once hypertension provides the necessary vascular substrate, atherogenic lipoprotein remodeling—rather than insulin-driven dysglycemia alone—is the dominant mechanistic axis driving all-cause mortality. From a clinical perspective, AIP is calculated from two routine lipid measurements and is therefore especially attractive for resource-limited rural primary-care settings, where it could be integrated into existing hypertension-management workflows to identify hypertensive patients who may benefit most from intensified lipid-modifying therapy.

A plausible explanation for the null finding in normotensive individuals is that, in the absence of sustained hemodynamic stress, key pressure-amplified pathways—endothelial dysfunction, arterial stiffening, and renin–angiotensin–aldosterone activation—remain largely quiescent. Without these amplifiers, the comparatively modest atherogenic and insulin-resistance signals captured by AIP and TyG are insufficient to translate into a detectable mortality difference over a 10-year follow-up, whereas the same indices become prognostically informative once hypertension provides the necessary vascular substrate.

## Limitations

Several limitations of this study should be acknowledged. First, the cohort was derived exclusively from a low-income rural population in northern China, which may limit the generalizability of our findings to urban residents or populations with different socioeconomic and ethnic backgrounds. Second, some lifestyle factors—including diet, physical activity, and genetic predisposition—were not comprehensively assessed, which may have introduced residual confounding and partially affected the accuracy of the observed associations between AIP, TyG, and all-cause mortality. Third, information on smoking and alcohol use was obtained through self-report, which may be subject to recall and social desirability bias, potentially leading to exposure misclassification. Fourth, although we adjusted for multiple conventional risk factors, unmeasured or unknown variables could still have influenced the associations. Finally, the study design was observational, precluding causal inference despite the prospective follow-up and rigorous statistical analyses. Future studies should include more diverse populations, incorporate objective and longitudinal assessments of lifestyle and genetic factors, and apply advanced causal inference approaches to validate and extend these findings.

## Conclusion

In this 10-year rural cohort from northern China, AIP and its modified indices showed a non-linear inverse association with all-cause mortality among hypertensive individuals, but no such association in the non-hypertensive stratum. AIP-based indices outperformed TyG-based indices for predicting all-cause mortality in hypertension. These findings support BP status as an effect modifier of lipid-ratio prognostic value and identify AIP as a low-cost candidate for refining residual cardiovascular risk stratification in hypertensive rural Chinese adults. Further validation in independent and more diverse populations is warranted before extrapolating these results to broader settings.

## Data Availability

The raw data supporting the conclusions of this article will be made available by the authors, without undue reservation.

## References

[1] Tan SCW Zheng BB Tang ML Chu H Zhao YT Weng C. Global burden of cardiovascular diseases and its risk factors, 1990-2021: a systematic analysis for the global burden of disease study 2021. QJM. (2025) 118(6):411–22. 10.1093/qjmed/hcaf022 39847534

[2] Chong B Jayabaskaran J Jauhari SM Chan SP Goh R Kueh MTW. Global burden of cardiovascular diseases: projections from 2025 to 2050. Eur J Prev Cardiol. (2025) 32(11):1001–15. 10.1093/eurjpc/zwae281 39270739

[3] Li M Chi X Wang Y Setrerrahmane S Xie W Xu H. Trends in insulin resistance: insights into mechanisms and therapeutic strategy. Signal Transduct Target Ther. (2022) 7(1):216. 10.1038/s41392-022-01073-0 35794109 PMC9259665

[4] Zheng X Zhang X Han Y Hu H Cao C. Nonlinear relationship between atherogenic index of plasma and the risk of prediabetes: a retrospective study based on Chinese adults. Cardiovasc Diabetol. (2023) 22(1):205. 10.1186/s12933-023-01934-0 37563588 PMC10416492

[5] Liu J Kang J Liang P Song Z Li G Jin X. The association between triglyceride-glucose index and all-cause/cardiovascular mortality in patients with different glucose metabolism statuses. Cardiovasc Diabetol. (2025) 24(1):367. 10.1186/s12933-025-02826-1 40993712 PMC12462038

[6] Cui H Liu Q Wu Y Cao L. Cumulative triglyceride-glucose index is a risk for CVD: a prospective cohort study. Cardiovasc Diabetol. (2022) 21(1):22. 10.1186/s12933-022-01456-1 35144621 PMC8830002

[7] Wu X Qiu W Yang H Chen YJ Liu J Zhao G. Associations of the triglyceride-glucose index and atherogenic index of plasma with the severity of new-onset coronary artery disease in different glucose metabolic states. Cardiovasc Diabetol. (2024) 23(1):76. 10.1186/s12933-024-02163-9 38378553 PMC10880297

[8] Xu S Zhang Z Li J Ding Y Chen Y Zhou Y. Does diabetes status modify the association between the triglyceride-glucose index and major adverse cardiovascular events in patients with coronary heart disease? A systematic review and meta-analysis of longitudinal cohort studies. Cardiovasc Diabetol. (2025) 24(1):317. 10.1186/s12933-025-02890-7 40760488 PMC12323116

[9] Powell-Wiley TM Poirier P Burke LE Després JP Gordon-Larsen P Lavie CJ. Obesity and cardiovascular disease: a scientific statement from the American Heart Association. Circulation. (2021) 143(21):e984–1010. 10.1161/CIR.0000000000000973 33882682 PMC8493650

[10] Qiao T Luo T Pei H Yimingniyazi B Aili D Aimudula A. Association between abdominal obesity indices and risk of cardiovascular events in Chinese populations with type 2 diabetes: a prospective cohort study. Cardiovasc Diabetol. (2022) 21(1):225. 10.1186/s12933-022-01670-x 36320060 PMC9628026

[11] Wu X Wang C Lv D Chen B Wu Y Wu X. Associations between Chinese visceral adiposity index and risks of all-cause and cause-specific mortality: a population-based cohort study. Diabetes Obes Metab. (2024) 26(4):1264–72. 10.1111/dom.15424 38164799

[12] Umemura S Arima H Arima S Asayama K Dohi Y Hirooka Y. The Japanese society of hypertension guidelines for the management of hypertension (JSH 2019). Hypertens Res. (2019) 42(9):1235–481. 10.1038/s41440-019-0284-9 31375757

[13] Touyz RM. New insights into mechanisms of hypertension. Curr Opin Nephrol Hypertens. (2012) 21(2):119–21. 10.1097/MNH.0b013e328350a50f 22257800

[14] World Health Organization. Obesity: preventing and managing the global epidemic. Report of a WHO consultation. World Health Organ Tech Rep Ser. (2000) 894:i–xii; 1–253. 11234459

[15] Hao J Chen R Abudukeremu D Li X Zhang Y Wang L. TyG-ABSI as a novel metabolic obesity indicator for carotid plaque: an explainable machine learning study using SHAP in low-income population. BMC Endocr Disord. (2025) 25(1):281. 10.1186/s12902-025-02099-5 41402834 PMC12709704

[16] Unger T Borghi C Charchar F Khan NA Poulter NR Prabhakaran D. 2020 International society of hypertension global hypertension practice guidelines. Hypertension. (2020) 75(6):1334–57. 10.1161/HYPERTENSIONAHA.120.15026 32370572

[17] American Diabetes Association. 2. Classification and diagnosis of diabetes: standards of medical care in diabetes-2021. Diabetes Care. (2021) 44(Suppl 1):S15–33. 10.2337/dc21-S002 33298413

[18] Simental-Mendía LE Rodríguez-Morán M Guerrero-Romero F. The product of fasting glucose and triglycerides as surrogate for identifying insulin resistance in apparently healthy subjects. Metab Syndr Relat Disord. (2008) 6(4):299–304. 10.1089/met.2008.0034 19067533

[19] Dobiásová M Frohlich J. The plasma parameter log (TG/HDL-C) as an atherogenic index: correlation with lipoprotein particle size and esterification rate in apoB-lipoprotein-depleted plasma (FER(HDL)). Clin Biochem. (2001) 34(7):583–8. 10.1016/S0009-9120(01)00263-6 11738396

[20] Park Y Kim NH Kwon TY Kim SG. A novel adiposity index as an integrated predictor of cardiometabolic disease morbidity and mortality. Sci Rep. (2018) 8(1):16753. 10.1038/s41598-018-35073-4 30425288 PMC6233180

[21] Yu Y Gu M Huang H Cheng S Deng Y Cai C. Combined association of triglyceride-glucose index and systolic blood pressure with all-cause and cardiovascular mortality among the general population. J Transl Med. (2022) 20(1):478. 10.1186/s12967-022-03678-z 36266665 PMC9583494

[22] Xu JP Peng XQ Guo LH Zhao XJ Chen MS Mai XY. The associations of the triglyceride-glucose index and its combination with blood pressure on cardiovascular and all-cause mortality in hypertension: a national study. Front Endocrinol. (2024) 15:1469055. 10.3389/fendo.2024.1469055 PMC11513261 39469572

[23] Ding J Li J Cai X Zhang K Yu S Liu K. The relationship between the triglyceride-glucose (TyG) index and all-cause mortality in ICU patients with primary hypertension: a retrospective study. Sci Rep. (2025) 15(1):12071. 10.1038/s41598-025-96202-4 40199939 PMC11978879

[24] Zhou D Liu XC Kenneth L Huang YQ Feng YQ. A non-linear association of triglyceride glycemic index with cardiovascular and all-cause mortality among patients with hypertension. Front Cardiovasc Med. (2021) 8:778038. 10.3389/fcvm.2021.778038 35155598 PMC8828937

[25] Tao LC Xu JN Wang TT Hua F Li JJ. Triglyceride-glucose index as a marker in cardiovascular diseases: landscape and limitations. Cardiovasc Diabetol. (2022) 21(1):68. 10.1186/s12933-022-01511-x 35524263 PMC9078015

[26] da Silva AA do Carmo JM Li X Wang Z Mouton AJ Hall JE. Role of hyperinsulinemia and insulin resistance in hypertension: metabolic syndrome revisited. Can J Cardiol. (2020) 36(5):671–82. 10.1016/j.cjca.2020.02.066 32389340 PMC7219403

[27] Tagi VM Mainieri F Chiarelli F. Hypertension in patients with insulin resistance: etiopathogenesis and management in children. Int J Mol Sci. (2022) 23(10):5814. 10.3390/ijms23105814 35628624 PMC9144705

[28] Steinberg HO Chaker H Leaming R Johnson A Brechtel G Baron AD. Obesity/insulin resistance is associated with endothelial dysfunction. Implications for the syndrome of insulin resistance. J Clin Invest. (1996) 97(11):2601–10. 10.1172/JCI118709 8647954 PMC507347

[29] Mancusi C Izzo R di Gioia G Losi MA Barbato E Morisco C. Insulin resistance the hinge between hypertension and type 2 diabetes. High Blood Press Cardiovasc Prev. (2020) 27(6):515–26. 10.1007/s40292-020-00408-8 32964344 PMC7661395

[30] Stegenga ME van der Crabben SN Levi M de Vos AF Tanck MW Sauerwein HP. Hyperglycemia stimulates coagulation, whereas hyperinsulinemia impairs fibrinolysis in healthy humans. Diabetes. (2006) 55(6):1807–12. 10.2337/db05-1543 16731846

[31] Dunn EJ Philippou H Ariëns RA Grant PJ. Molecular mechanisms involved in the resistance of fibrin to clot lysis by plasmin in subjects with type 2 diabetes mellitus. Diabetologia. (2006) 49(5):1071–80. 10.1007/s00125-006-0197-4 16538489

[32] Wang C Du Z Ye N Liu S Geng D Wang P. Using the atherogenic index of plasma to estimate the prevalence of ischemic stroke within a general population in a rural area of China. Biomed Res Int. (2020) 2020:7197054. 10.1155/2020/7197054 33490253 PMC7787721

[33] You FF Gao J Gao YN Li ZH Shen D Zhong WF. Association between atherogenic index of plasma and all-cause mortality and specific-mortality: a nationwide population-based cohort study. Cardiovasc Diabetol. (2024) 23(1):276. 10.1186/s12933-024-02370-4 39068437 PMC11283706

[34] Zheng Q Cao Z Teng J Lu Q Huang P Zhou J. Association between atherogenic index of plasma with all-cause and cardiovascular mortality in individuals with cardiovascular-kidney-metabolic syndrome. Cardiovasc Diabetol. (2025) 24(1):183. 10.1186/s12933-025-02742-4 40287685 PMC12034140

[35] Duiyimuhan G Maimaiti N. The association between atherogenic index of plasma and all-cause mortality and cardiovascular disease-specific mortality in hypertension patients: a retrospective cohort study of NHANES. BMC Cardiovasc Disord. (2023) 23(1):452. 10.1186/s12872-023-03451-0 37697281 PMC10496369

[36] Qian J Chi Q Qian C Fan X Ding W Wang T. Atherogenic index of plasma and triglyceride-glucose index mediate the association between stroke and all-cause mortality: insights from the lipid paradox. Lipids Health Dis. (2025) 24(1):173. 10.1186/s12944-025-02586-7 40349063 PMC12065248

[37] Dobiásová M Frohlich J Sedová M Cheung MC Brown BG. Cholesterol esterification and atherogenic index of plasma correlate with lipoprotein size and findings on coronary angiography. J Lipid Res. (2011) 52(3):566–71. 10.1194/jlr.P011668 21224290 PMC3035693

