# Supplementary material for: Blood pressure–stratified associations of the atherogenic index of plasma with all-cause mortality: a 10-year rural cohort study in China
Source: Front Cardiovasc Med. 2026 Jun 8;13:1747068. doi: 10.3389/fcvm.2026.1747068 (PMC13283828; doi:10.3389/fcvm.2026.1747068)
Supplement: Supplementary file 1 [file Datasheet1.docx]

Supplementary Table 1. Number of participants at risk at each follow-up time point for the Kaplan–Meier survival curves shown in Figure 2 (Hypertension group).*

| Indicator | Quartile | Follow-up time (years) | | | | |
| --- | --- | --- | --- | --- | --- | --- |
|  |  | 0 | 2.5 | 5 | 7.5 | 10 |
| TyG | Q1 (low) | 670 | 624 | 548 | 402 | 247 |
| TyG | Q2 | 661 | 610 | 532 | 442 | 385 |
| TyG | Q3 | 666 | 647 | 618 | 579 | 547 |
| TyG | Q4 (high) | 665 | 647 | 615 | 586 | 552 |
| TyG_BMI | Q1 (low) | 665 | 622 | 546 | 422 | 298 |
| TyG_BMI | Q2 | 664 | 626 | 557 | 465 | 388 |
| TyG_BMI | Q3 | 664 | 636 | 599 | 543 | 504 |
| TyG_BMI | Q4 (high) | 665 | 640 | 607 | 575 | 537 |
| TyG_WHtR | Q1 (low) | 665 | 626 | 565 | 438 | 317 |
| TyG_WHtR | Q2 | 664 | 634 | 562 | 471 | 403 |
| TyG_WHtR | Q3 | 664 | 626 | 582 | 535 | 486 |
| TyG_WHtR | Q4 (high) | 665 | 638 | 600 | 561 | 521 |
| TyG_WC | Q1 (low) | 665 | 631 | 570 | 438 | 314 |
| TyG_WC | Q2 | 664 | 621 | 545 | 466 | 385 |
| TyG_WC | Q3 | 664 | 627 | 583 | 525 | 492 |
| TyG_WC | Q4 (high) | 665 | 645 | 611 | 576 | 536 |
| TyG_WWI | Q1 (low) | 665 | 633 | 571 | 452 | 332 |
| TyG_WWI | Q2 | 664 | 628 | 564 | 481 | 424 |
| TyG_WWI | Q3 | 664 | 623 | 579 | 521 | 471 |
| TyG_WWI | Q4 (high) | 665 | 640 | 595 | 551 | 500 |
| AIP | Q1 (low) | 666 | 653 | 545 | 336 | 153 |
| AIP | Q2 | 665 | 575 | 506 | 475 | 440 |
| AIP | Q3 | 665 | 649 | 631 | 594 | 563 |
| AIP | Q4 (high) | 666 | 651 | 631 | 604 | 575 |
| AIP_BMI | Q1 (low) | 665 | 643 | 541 | 337 | 152 |
| AIP_BMI | Q2 | 664 | 583 | 508 | 472 | 438 |
| AIP_BMI | Q3 | 664 | 647 | 629 | 592 | 559 |
| AIP_BMI | Q4 (high) | 665 | 651 | 631 | 604 | 578 |
| AIP_WHtR | Q1 (low) | 665 | 649 | 542 | 336 | 152 |
| AIP_WHtR | Q2 | 664 | 577 | 507 | 473 | 439 |
| AIP_WHtR | Q3 | 664 | 648 | 631 | 594 | 562 |
| AIP_WHtR | Q4 (high) | 665 | 650 | 629 | 602 | 574 |
| AIP_WC | Q1 (low) | 665 | 649 | 545 | 336 | 152 |
| AIP_WC | Q2 | 664 | 577 | 504 | 473 | 439 |
| AIP_WC | Q3 | 664 | 647 | 629 | 591 | 561 |
| AIP_WC | Q4 (high) | 665 | 651 | 631 | 605 | 575 |
| AIP_WWI | Q1 (low) | 665 | 651 | 546 | 337 | 152 |
| AIP_WWI | Q2 | 664 | 575 | 503 | 472 | 439 |
| AIP_WWI | Q3 | 664 | 648 | 631 | 593 | 564 |
| AIP_WWI | Q4 (high) | 665 | 650 | 629 | 603 | 572 |

*Abbreviations: Each group was divided into four intervals based on the 25th, 50th, and 75th percentiles of the group's sample, with each interval expressed as [lower limit – upper limit]: Q1 = minimum value to P25, Q2 = P25 to P50 (median), Q3 = P50 to P75, and Q4 = P75 to maximum value. All numerical values are rounded to two decimal places. TyG, triglyceride-glucose index; AIP, atherogenic index of plasma; BMI, body mass index; WC, waist circumference; WHtR, waist-to-height ratio; WWI, weight-adjusted waist index.

Supplementary Table 2. Number of participants at risk at each follow-up time point for the Kaplan–Meier survival curves shown in Figure 3 (Non-hypertension group).*

| Indicator | Quartile | Follow-up time (years) | | | | |
| --- | --- | --- | --- | --- | --- | --- |
|  |  | 0 | 2.5 | 5 | 7.5 | 10 |
| TyG | Q1 (low) | 316 | 313 | 309 | 302 | 298 |
| TyG | Q2 | 315 | 310 | 303 | 295 | 285 |
| TyG | Q3 | 315 | 308 | 304 | 296 | 292 |
| TyG | Q4 (high) | 316 | 313 | 309 | 300 | 294 |
| TyG_BMI | Q1 (low) | 315 | 307 | 300 | 286 | 278 |
| TyG_BMI | Q2 | 314 | 312 | 306 | 301 | 295 |
| TyG_BMI | Q3 | 314 | 308 | 306 | 301 | 294 |
| TyG_BMI | Q4 (high) | 315 | 314 | 310 | 302 | 299 |
| TyG_WHtR | Q1 (low) | 315 | 311 | 308 | 299 | 296 |
| TyG_WHtR | Q2 | 314 | 307 | 300 | 294 | 284 |
| TyG_WHtR | Q3 | 314 | 311 | 307 | 299 | 292 |
| TyG_WHtR | Q4 (high) | 315 | 312 | 307 | 298 | 294 |
| TyG_WC | Q1 (low) | 315 | 310 | 308 | 299 | 296 |
| TyG_WC | Q2 | 314 | 307 | 296 | 289 | 279 |
| TyG_WC | Q3 | 314 | 309 | 307 | 303 | 297 |
| TyG_WC | Q4 (high) | 315 | 315 | 311 | 299 | 294 |
| TyG_WWI | Q1 (low) | 315 | 312 | 311 | 306 | 303 |
| TyG_WWI | Q2 | 314 | 309 | 305 | 298 | 292 |
| TyG_WWI | Q3 | 314 | 308 | 302 | 294 | 285 |
| TyG_WWI | Q4 (high) | 315 | 312 | 304 | 292 | 286 |
| AIP | Q1 (low) | 316 | 311 | 304 | 296 | 291 |
| AIP | Q2 | 316 | 311 | 308 | 300 | 292 |
| AIP | Q3 | 314 | 309 | 303 | 293 | 287 |
| AIP | Q4 (high) | 316 | 313 | 310 | 304 | 299 |
| AIP_BMI | Q1 (low) | 315 | 310 | 303 | 294 | 289 |
| AIP_BMI | Q2 | 314 | 309 | 306 | 299 | 291 |
| AIP_BMI | Q3 | 314 | 310 | 305 | 295 | 289 |
| AIP_BMI | Q4 (high) | 315 | 312 | 308 | 302 | 297 |
| AIP_WHtR | Q1 (low) | 315 | 310 | 303 | 293 | 288 |
| AIP_WHtR | Q2 | 314 | 309 | 306 | 300 | 292 |
| AIP_WHtR | Q3 | 314 | 310 | 305 | 295 | 289 |
| AIP_WHtR | Q4 (high) | 315 | 312 | 308 | 302 | 297 |
| AIP_WC | Q1 (low) | 315 | 310 | 303 | 293 | 288 |
| AIP_WC | Q2 | 314 | 309 | 306 | 300 | 292 |
| AIP_WC | Q3 | 314 | 310 | 304 | 294 | 288 |
| AIP_WC | Q4 (high) | 315 | 312 | 309 | 303 | 298 |
| AIP_WWI | Q1 (low) | 315 | 309 | 301 | 292 | 287 |
| AIP_WWI | Q2 | 314 | 310 | 308 | 301 | 293 |
| AIP_WWI | Q3 | 314 | 310 | 306 | 296 | 290 |
| AIP_WWI | Q4 (high) | 315 | 312 | 307 | 301 | 296 |

*Abbreviations: Each group was divided into four intervals based on the 25th, 50th, and 75th percentiles of the group's sample, with each interval expressed as [lower limit – upper limit]: Q1 = minimum value to P25, Q2 = P25 to P50 (median), Q3 = P50 to P75, and Q4 = P75 to maximum value. All numerical values are rounded to two decimal places. TyG, triglyceride-glucose index; AIP, atherogenic index of plasma; BMI, body mass index; WC, waist circumference; WHtR, waist-to-height ratio; WWI, weight-adjusted waist index.

Supplementary Table 3. Distribution of quartile intervals (Q1–Q4) for each combined index in the hypertensive group and non-hypertensive group.*

| Indices | Groups | Q1 | Q2 | Q3 | Q4 |
| --- | --- | --- | --- | --- | --- |
| TyG | Non-Hypertensive Group | 6.79 – 8.30 | 8.30 – 8.64 | 8.64 – 9.09 | 9.09 – 11.31 |
|  | Hypertensive Group | 6.92 – 8.44 | 8.44 – 8.82 | 8.82 – 9.27 | 9.27 – 12.00 |
| AIP | Non-Hypertensive Group | -1.95 – -0.51 | -0.51 – -0.10 | -0.10 – 0.44 | 0.44 – 3.02 |
|  | Hypertensive Group | -2.41 – -0.41 | -0.41 – 0.06 | 0.06 – 0.58 | 0.58 – 2.90 |
| TyGWWI | Non-Hypertensive Group | 64.01 – 88.47 | 88.47 – 94.60 | 94.60 – 102.20 | 102.20 – 143.81 |
|  | Hypertensive Group | 69.11 – 91.84 | 91.84 – 98.51 | 98.51 – 105.67 | 105.67 – 144.73 |
| TyGWC | Non-Hypertensive Group | 461.60 – 679.99 | 679.99 – 753.27 | 753.27 – 826.65 | 826.65 – 1180.31 |
|  | Hypertensive Group | 493.51 – 727.38 | 727.38 – 798.10 | 798.10 – 876.81 | 876.81 – 1332.70 |
| TyGBMI | Non-Hypertensive Group | 120.81 – 188.93 | 188.93 – 211.95 | 211.95 – 238.29 | 238.29 – 397.77 |
|  | Hypertensive Group | 122.59 – 204.42 | 204.42 – 228.25 | 228.25 – 258.00 | 258.00 – 413.18 |
| TyGWHtR | Non-Hypertensive Group | 3.02 – 4.26 | 4.26 – 4.70 | 4.70 – 5.17 | 5.17 – 7.61 |
|  | Hypertensive Group | 2.97 – 4.56 | 4.56 – 5.01 | 5.01 – 5.51 | 5.51 – 8.60 |
| AIPWHtR | Non-Hypertensive Group | -0.92 – -0.27 | -0.27 – -0.05 | -0.05 – 0.25 | 0.25 – 1.70 |
|  | Hypertensive Group | -1.47 – -0.23 | -0.23 – 0.03 | 0.03 – 0.33 | 0.33 – 1.68 |
| AIPWC | Non-Hypertensive Group | -146.43 – -43.60 | -43.60 – -7.83 | -7.83 – 39.01 | 39.01 – 293.31 |
|  | Hypertensive Group | -221.26 – -36.15 | -36.15 – 5.54 | 5.54 – 52.96 | 52.96 – 301.34 |
| AIPWWI | Non-Hypertensive Group | -19.65 – -5.57 | -5.57 – -1.02 | -1.02 – 4.89 | 4.89 – 31.81 |
|  | Hypertensive Group | -28.10 – -4.56 | -4.56 – 0.72 | 0.72 – 6.49 | 6.49 – 31.25 |
| AIPBMI | Non-Hypertensive Group | -44.07 – -12.00 | -12.00 – -2.24 | -2.24 – 11.35 | 11.35 – 85.88 |
|  | Hypertensive Group | -65.40 – -10.22 | -10.22 – 1.58 | 1.58 – 15.58 | 15.58 – 84.10 |

*Each group was divided into four intervals based on the 25th, 50th, and 75th percentiles of the group's sample, with each interval expressed as [lower limit – upper limit]: Q1 = minimum value to P25, Q2 = P25 to P50 (median), Q3 = P50 to P75, and Q4 = P75 to maximum value. All numerical values are rounded to two decimal places. TyG = Triglyceride-Glucose Index; AIP = Atherosclerosis Index; BMI = Body Mass Index; WC = Waist Circumference; WHtR = Waist Circumference-to-Height Ratio; WWI = Weight-Adjusted Waist Circumference Index.

Supplementary Table 4. Subgroup Analysis Stratified by Sex.*

| Characteristics | Men  HR (95% CI) | P | Women  HR (95% CI) | P |
| --- | --- | --- | --- | --- |
| Hypertensive Group： |  |  |  |  |
| TyG | 0.49 (0.41, 0.58) | <0.001 | 0.41 (0.35, 0.47) | <0.001 |
| TyG-BMI | 0.99 (0.99, 0.99) | <0.001 | 0.99 (0.99, 0.99) | <0.001 |
| TyG-WHtR | 0.59 (0.50, 0.69) | <0.001 | 0.59 (0.52, 0.67) | <0.001 |
| TyG-WC | 1.00 (1.00, 1.00) | <0.001 | 1.00 (1.00, 1.00) | <0.001 |
| TyG-WWI | 0.97 (0.96, 0.98) | <0.001 | 0.96 (0.95, 0.97) | <0.001 |
| AIP | 0.49 (0.42, 0.56) | <0.001 | 0.38 (0.34, 0.43) | <0.001 |
| AIP-BMI | 0.97 (0.97, 0.98) | <0.001 | 0.96 (0.96, 0.97) | <0.001 |
| AIP-WHtR | 0.26 (0.19, 0.34) | <0.001 | 0.19 (0.15, 0.23) | <0.001 |
| AIP-WC | 0.99 (0.99, 0.99) | <0.001 | 0.99 (0.99, 0.99) | <0.001 |
| AIP-WWI | 0.94 (0.92, 0.95) | <0.001 | 0.92 (0.91, 0.93) | <0.001 |
| Non-Hypertensive Group: |  |  |  |  |
| TyG | 1.07 (0.63, 1.81) | 0.816 | 1.31 (0.70, 2.44) | 0.403 |
| TyG-BMI | 1.00 (0.99, 1.01) | 0.631 | 1.00 (0.99, 1.01) | 0.861 |
| TyG-WHtR | 0.91 (0.56, 1.48) | 0.705 | 1.18 (0.71, 1.96) | 0.513 |
| TyG-WC | 1.00 (1.00, 1.00) | 0.453 | 1.00 (1.00, 1.00) | 0.595 |
| TyG-WWI | 1.00 (0.97, 1.03) | 0.951 | 1.02 (0.99, 1.05) | 0.297 |
| AIP | 0.90 (0.60, 1.36) | 0.625 | 0.88 (0.52, 1.46) | 0.615 |
| AIP-BMI | 1.00 (0.98, 1.01) | 0.687 | 1.00 (0.98, 1.02) | 0.754 |
| AIP-WHtR | 0.82 (0.37, 1.80) | 0.619 | 0.80 (0.31, 2.05) | 0.640 |
| AIP-WC | 1.00 (0.99, 1.00) | 0.663 | 1.00 (0.99, 1.01) | 0.656 |
| AIP-WWI | 0.99 (0.95, 1.03) | 0.592 | 0.99 (0.94, 1.03) | 0.584 |

*Adjusted Covariates: Age, years of education, smoking history, and alcohol consumption history. Abbreviations: HR, hazard ratio; CI, confidence interval; ; TyG, triglyceride-glucose index; AIP, atherogenic index of plasma; BMI, body mass index; WC, waist circumference; WHtR, waist-to-height ratio; WWI, weight-adjusted waist index.

Supplementary Table 5. Subgroup Analysis Stratified by Age Groups.*

| Characteristics | <60 Group  HR (95% CI) | P | ≥60 Group  HR (95% CI) | P |
| --- | --- | --- | --- | --- |
| Hypertensive Group： |  |  |  |  |
| TyG | 0.30 (0.25, 0.36) | <0.001 | 0.54 (0.47, 0.61) | <0.001 |
| TyG-BMI | 0.99 (0.98, 0.99) | <0.001 | 0.99 (099, 0.99) | <0.001 |
| TyG-WHtR | 0.43 (0.36, 0.52) | <0.001 | 0.68 (0.61, 0.77) | <0.001 |
| TyG-WC | 1.00 (0.99, 1.00) | <0.001 | 1.00 (1.00, 1.00) | <0.001 |
| TyG-WWI | 0.94 (0.92, 0.95) | <0.001 | 0.98 (0.97, 0.99) | <0.001 |
| AIP | 0.31 (0.27, 0.36) | <0.001 | 0.49 (0.44, 0.55) | <0.001 |
| AIP-BMI | 0.95 (0.95, 0.96) | <0.001 | 0.97 (0.97, 0.98) | <0.001 |
| AIP-WHtR | 0.12 (0.09, 0.16) | <0.001 | 0.27 (0.22, 0.33) | <0.001 |
| AIP-WC | 0.99 (0.99, 0.99) | <0.001 | 0.99 (0.99, 0.99) | <0.001 |
| AIP-WWI | 0.90 (0.89, 0.91) | <0.001 | 0.94 (0.93, 0.95) | <0.001 |
| Non-Hypertensive Group: |  |  |  |  |
| TyG | 2.43 (1.18, 4.98) | 0.016 | 0.94 (0.62, 1.41) | 0.757 |
| TyG-BMI | 1.00 (0.98, 1.01) | 0.614 | 1.00 (0.99, 1.00) | 0.233 |
| TyG-WHtR | 1.14 (0.52, 2.51) | 0.750 | 1.04 (0.74, 1.47) | 0.815 |
| TyG-WC | 1.00 (1.00, 1.01) | 0.665 | 1.00 (1.00, 1.00) | 0.516 |
| TyG-WWI | 1.05 (1.00, 1.10) | 0.071 | 1.02 (0.99, 1.04) | 0.177 |
| AIP | 1.70 (0.89, 3.24) | 0.110 | 0.80 (0.57, 1.13) | 0.205 |
| AIP-BMI | 1.02 (0.99, 1.04) | 0.155 | 0.99 (0.98, 1.01) | 0.241 |
| AIP-WHtR | 2.45 (0.75, 8.03) | 0.140 | 0.62 (0.33, 1.18) | 0.148 |
| AIP-WC | 1.01 (1.00, 1.01) | 0.146 | 1.00 (0.99, 1.00) | 0.177 |
| AIP-WWI | 1.05 (0.99, 1.12) | 0.119 | 0.98 (0.95, 1.01) | 0.130 |

*Adjusted Covariates: Sex, years of education, smoking history, and alcohol consumption history. Abbreviations: HR, hazard ratio; CI, confidence interval; ; TyG, triglyceride-glucose index; AIP, atherogenic index of plasma; BMI, body mass index; WC, waist circumference; WHtR, waist-to-height ratio; WWI, weight-adjusted waist index.

Supplementary Table 6. Subgroup Analysis Stratified by Diabetes Status.*

| Characteristics | Diabetic Group  HR (95%) | P | Non-Diabetic Group  HR (95%) | P |
| --- | --- | --- | --- | --- |
| Hypertensive Group： |  |  |  |  |
| TyG | 0.51 (0.41, 0.64) | <0.001 | 0.38 (0.33, 0.44) | <0.001 |
| TyG-BMI | 0.99 (0.99, 1.00) | <0.001 | 0.99 (0.99, 0.99) | <0.001 |
| TyG-WHtR | 0.61 (0.50, 0.75) | <0.001 | 0.56 (0.49, 0.63) | <0.001 |
| TyG-WC | 1.00 (1.00, 1.00) | <0.001 | 1.00 (1.00, 1.00) | <0.001 |
| TyG-WWI | 0.97 (0.95, 0.98) | <0.001 | 0.96 (0.95, 0.97) | <0.001 |
| AIP | 0.45 (0.37, 0.55) | <0.001 | 0.41 (0.37, 0.46) | <0.001 |
| AIP-BMI | 0.97 (0.96, 0.98) | <0.001 | 0.96 (0.96, 0.97) | <0.001 |
| AIP-WHtR | 0.25 (0.18, 0.36) | <0.001 | 0.19 (0.16, 0.24) | <0.001 |
| AIP-WC | 0.99 (0.99, 0.99) | <0.001 | 0.99 (0.99, 0.99) | <0.001 |
| AIP-WWI | 0.93 (0.92, 0.95) | <0.001 | 0.92 (0.91, 0.93) | <0.001 |
| Non-Hypertensive Group: |  |  |  |  |
| TyG | 1.08 (0.48, 2.44) | 0.855 | 0.77 (0.45, 1.29) | 0.316 |
| TyG-BMI | 1.00 (0.99, 1.01) | 0.488 | 1.00 (0.99, 1.00) | 0.221 |
| TyG-WHtR | 0.79 (0.43, 1.45) | 0.441 | 0.85 (0.56, 1.30) | 0.461 |
| TyG-WC | 1.00 (0.99, 1.00) | 0.207 | 1.00 (1.00, 1.00) | 0.452 |
| TyG-WWI | 0.99 (0.94, 1.04) | 0.583 | 1.00 (0.97, 1.02) | 0.813 |
| AIP | 0.83 (0.42, 1.64) | 0.592 | 0.87 (0.59, 1.27) | 0.460 |
| AIP-BMI | 0.99 (0.96, 1.02) | 0.597 | 1.00 (0.98, 1.01) | 0.603 |
| AIP-WHtR | 0.70 (0.20, 2.47) | 0.575 | 0.75 (0.37, 1.53) | 0.428 |
| AIP-WC | 1.00 (0.99, 1.01) | 0.511 | 1.00 (0.99, 1.00) | 0.516 |
| AIP-WWI | 0.98 (0.92, 1.05) | 0.573 | 0.98 (0.95, 1.02) | 0.373 |

*Adjusted Covariates: Sex, age, years of education, smoking history, and alcohol consumption history. Abbreviations: HR, hazard ratio; CI, confidence interval; ; TyG, triglyceride-glucose index; AIP, atherogenic index of plasma; BMI, body mass index; WC, waist circumference; WHtR, waist-to-height ratio; WWI, weight-adjusted waist index.
